# Synchronous gastric cancer and carcinoma in situ of the pancreas

**DOI:** 10.1002/ccr3.4892

**Published:** 2021-10-04

**Authors:** Masashi Inoue, Keishi Hakoda, Hiroyuki Sawada, Ryuichi Hotta, Ichiro Ohmori, Kazuaki Miyamoto, Kazuhiro Toyota, Seiji Sadamoto, Tadateru Takahashi

**Affiliations:** ^1^ Department of Surgery National Hospital Organization Higashihiroshima Medical Center Higashihiroshima Japan; ^2^ Department of Gastrointestinal and Transplant Surgery Applied Life Sciences Institute of Biomedical and Health Sciences Hiroshima University Hiroshima Japan

**Keywords:** gastric cancer, pancreatic cancer, serial pancreatic juice aspiration cytological examination, Stage 0

## Abstract

Abnormal findings in the pancreatic duct without a mass may require serial pancreatic juice aspiration cytological examination. In cases of synchronous gastric cancer and stage 0 pancreatic cancer, spleen‐preserving pancreatectomy may have advantage.

## Graphical abstract

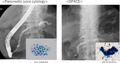

## 1 INTRODUCTION

Diagnosing pancreatic cancer at stage 0 can improve the prognosis. Abnormal findings in the pancreatic duct without a mass may require serial pancreatic juice aspiration cytological examination. In cases of synchronous gastric cancer, optimal selection of the surgical procedure in consideration of adjuvant chemotherapy is an issue for future evaluation.

The prognosis for patients with pancreatic cancer could be improved if clinicians were able to detect and treat the disease in its early stages.^1^, ^2^ Using a recent analysis of the Japan Pancreatic Cancer Registry, the Japan Pancreas Society reports that the 5‐year survival rate in patients with lesions less than 10 mm in diameter is 80.4%, and that it is 85.8% in patients with stage 0 pancreatic cancer, comprising high‐grade pancreatic intraepithelial neoplasia (PanIN‐3) and pancreatic carcinoma in situ (PCIS).^3^ However, only 0.6% of patients with pancreatic cancer are diagnosed at stage 0 because of the difficulty of detecting cancer at this early stage.^4^ We describe herein a patient with PCIS synchronous with early gastric cancer and detected preoperatively using serial pancreatic juice aspiration cytological examination (SPACE).

## 2 CASE REPORT

**FIGURE 1:**
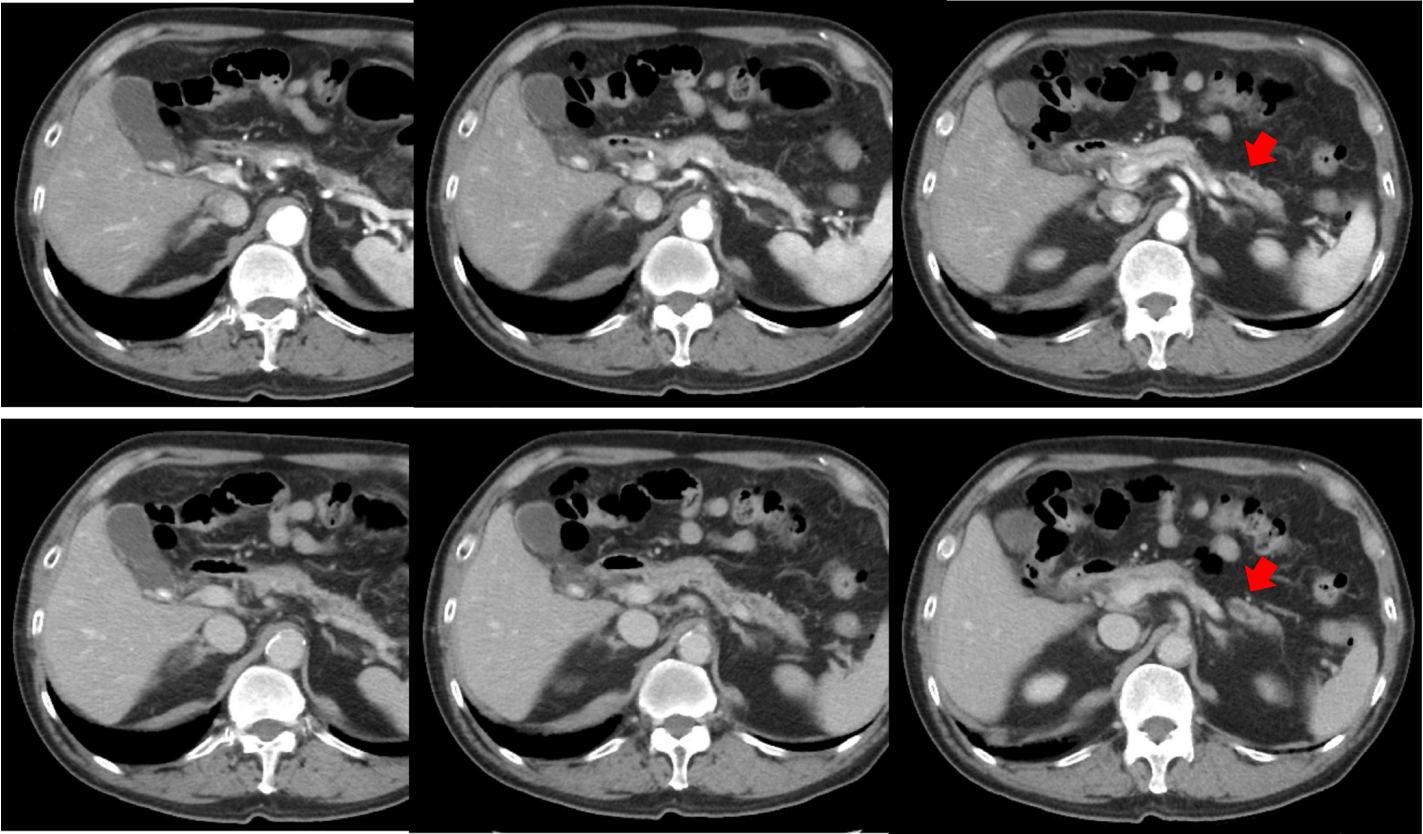
Computed tomography. The main pancreatic duct in the body and tail of the pancreas is dilated, but there is no visible tumor

**FIGURE 2:**
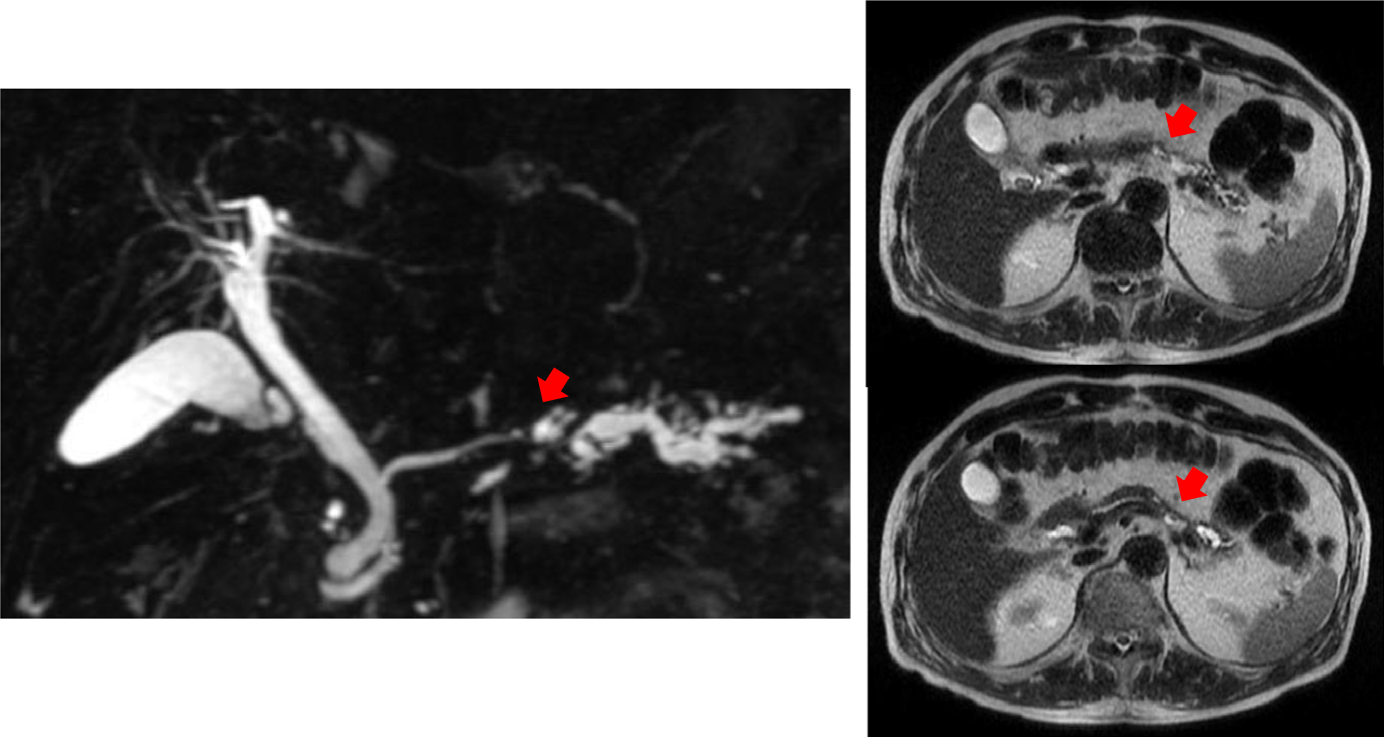
Magnetic resonance imaging. The main pancreatic duct in the body and tail of the pancreas is dilated, but there is no visible tumor

**FIGURE 3:**
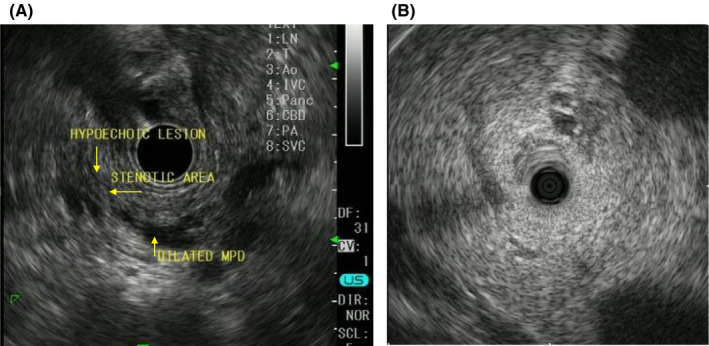
Endoscopic ultrasonography (A) and intraductal ultrasonography (B). The main pancreatic duct in the body and tail of the pancreas is dilated, but there is no visible tumor

**FIGURE 4:**
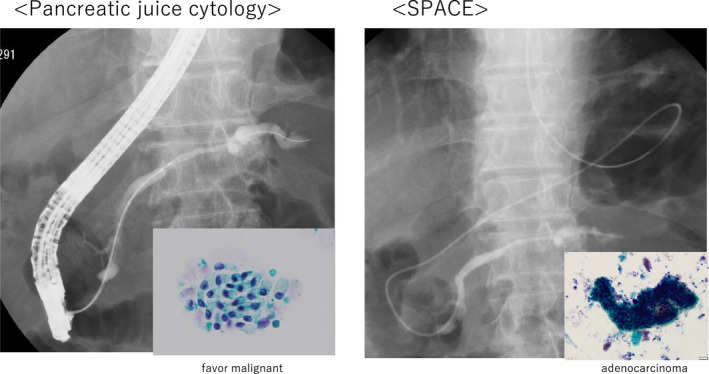
The results of pancreatic juice cytology favor malignancy but do not reveal adenocarcinoma

A 78‐year‐old man was hospitalized with upper abdominal pain. The medical history was unremarkable. Laboratory testing revealed no remarkable findings. The serum levels of carcinoembryonic antigen and carbohydrate antigen 19‐9 were 1.8 ng/ml and <2.0 U/ml, respectively. Gastrointestinal endoscopy showed a 0‐IIc lesion on the lesser curvature of the stomach, at the angulus with a depth consistent with submucosal invasion; biopsy revealed signet ring cell carcinoma. Abdominal computed tomography (CT) and magnetic resonance cholangiopancreatography (MRCP) showed that the main pancreatic duct in the body and tail of the pancreas was dilated, but neither scan revealed a lesion consistent with a tumor (Figures 1 and 2). Endoscopic ultrasonography (EUS) and intraductal ultrasonography revealed stenosis of the main pancreatic duct with dilation of the caudal pancreatic duct in the body of the pancreas, but they also did not show any mass (Figure 3). The results of endoscopic retrograde cholangiopancreatography (ERCP) with pancreatic juice cytology favored malignancy but did not reveal adenocarcinoma. We performed SPACE by placing a 4‐Fr αtype nasopancreatic tube (Gadelius Medical). Three of the 6 cytologic specimens revealed adenocarcinoma (Figure 4). The preoperative diagnosis was stage IA gastric cancer (cT1bN0M0) with stage 0 cancer of the pancreatic body (TisN0M0).

**FIGURE 5:**
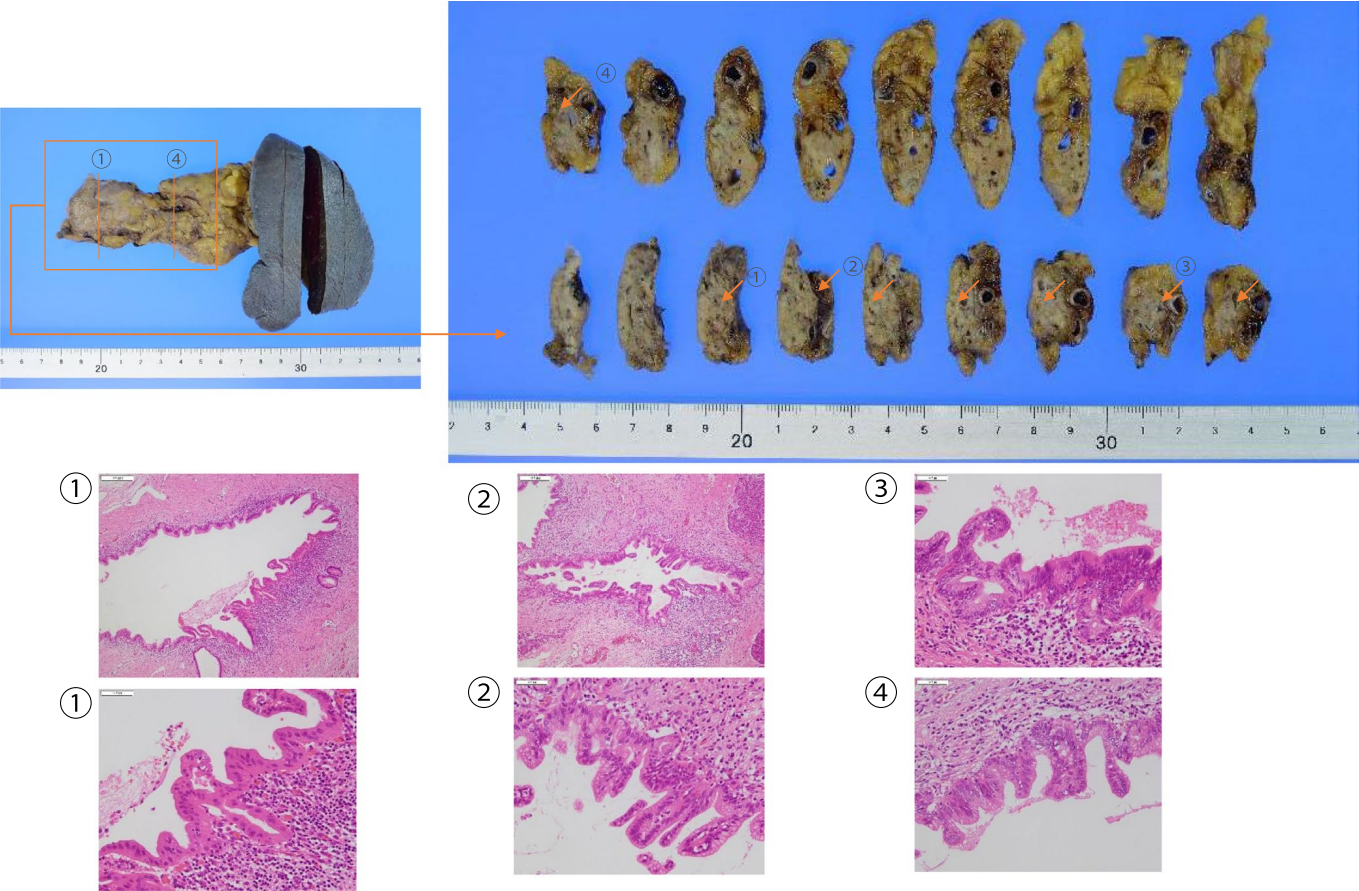
Postoperative pathologic examination. Pancreatic intraepithelial neoplasia grade 3 is seen in specimens 1 through 4, and the caudal pancreatic duct is dilated

We performed total gastrectomy and distal pancreatectomy with splenectomy. Histopathologic examination found PanIN‐3 with lymphocyte infiltration throughout the pancreas, including the tail, with dilation of the caudal pancreatic duct, the resection margins were negative for malignant cells. The postoperative diagnosis was stage 0 pancreatic body cancer (TisN0M0) and stage IA gastric cancer (T1bN0M0) (Figure 5).

The patient required enteral nutrition via a feeding tube for malnutrition after surgery. Two months after surgery, he was discharged in good general condition with a prescription for oral nutrition, although his history of malnutrition precluded the use of adjuvant chemotherapy. There was no evidence of recurrence at his most recent follow‐up appointment, 2 years after surgery.

## 3 DISCUSSION

The Japanese guideline for pancreatic cancer recommends ERCP if there are findings suggestive of pancreatic cancer, such as pancreatic duct dilation or stenosis, even if imaging does not show any evidence of a mass.^5^ Although no definitive pathologic diagnosis was obtained in our patient using pancreatic juice cytology obtained during ERCP, we were able to make the diagnosis using specimens obtained from SPACE. Iiboshi et al compared patients diagnosed using single pancreatic juice cytology versus SPACE and report that the sensitivity, specificity, and accuracy of SPACE are 100%, 83.3%, and 95%, respectively.^6^ When localized stenosis of the PD, a caliber change, and dilatation of the branch duct are demonstrated, ERCP followed by SPACE is recommended because of the high sensitivity. SPACE is considered essential for the diagnosis of PCIS.^5^

According to previous reports, patients with early pancreatic cancer are rarely symptomatic but frequently demonstrate focal branch‐duct dilation, focal irregular stenosis, small cystic lesions around the area of ductal stenosis, and distal dilation of the main pancreatic duct on EUS and MRCP.^4^ In patients with PCIS, assessment with ERCP frequently reveals irregularity, noncontinuous narrowing, granular defects, and dilation. Kikuyama et al report that patients with stage 0 disease demonstrate a high degree of fatty change on enhanced CT, in the pancreatic parenchyma around the area of PCIS.^7^ In certain patients with PCIS, focal pancreatitis with inflammatory cells, desmoplastic changes, fibrosis, and fatty changes are observed in the parenchyma around the area of PCIS.^8^, ^9^, ^10^ Ikeda et al classify PCIS into 3 types: flat, low papillary, and mixed.^11^ Based on this classification system, our patient's disease would be classified as the low papillary type, which may tend to spread intraductally. As PCIS with intraductal spread into the main pancreatic duct and the branch duct can cause focal pancreatitis, patients may experience stenosis and dilation of the main pancreatic duct. We confirmed the presence of lymphocyte infiltration in our patient, and we believe that his stenosis was triggered by inflammation.

In Japan, the use of an oral fluoropyrimidine (S‐1) is indicated for postoperative adjuvant chemotherapy in patients with pancreatic cancer.^12^ However, there is no evidence regarding the need for adjuvant chemotherapy for PCIS. More case reports are needed to determine whether adjuvant chemotherapy is indeed indicated for patients with PCIS.

Considering the risk‐benefit balance for each patient, S‐1 alone is recommended for Stage II, and S‐1 alone or an oxaliplatin combination, such as CapeOX, is recommended as adjuvant chemotherapy for stage III gastric cancer in Japan.^13^ As in our case, patients with both gastric cancer and pancreatic body cancer may require total gastrectomy with distal pancreatectomy, since distal gastrectomy with distal pancreatectomy may leave the patient with venous stasis and insufficient arterial perfusion to the remnant stomach. Surgical removal of the stomach and pancreas together can lead to a severe postoperative nutritional imbalance and derangement of glucose metabolism in patients.^14^ In contrast, a spleen‐preserving distal pancreatectomy with conservation of the splenic artery and vein for a pancreatic benign or low grade malignant pancreatic tumor is widely accepted.^13^ Based on a review of the PubMed database, no case of synchronous gastric cancer and PCIS has been reported. In this case, postoperative adjuvant chemotherapy was not possible due to poor postoperative nutritional status, but there was no evidence for stage 0 pancreatic and stageⅠgastric cancers. If distal pancreatectomy with spleen preservation is approved for stage 0 pancreatic cancer, distal gastrectomy is also indicated for stage II and III gastric cancer, and it is possible to prolong the prognosis by improving the postoperative nutritional status and introducing postoperative adjuvant chemotherapy for both tumors. Additional case reports are needed to determine whether the extent of lymph node dissection can be safely reduced for advantage of nutrition.

## 4 CONCLUSION

PCIS frequently presents as incidental pancreatic duct stenosis, and the addition of SPACE improves diagnostic sensitivity. The indications for adjuvant chemotherapy for stage 0 pancreatic cancer, and in cases of synchronous gastric cancer and stage 0 pancreatic cancer, whether spleen‐preserving distal gastrectomy is acceptable to facilitate optimal nutrition and adjuvant chemotherapy are unresolved issues.

## CONFLICTS OF INTEREST

There are no potential conflicts of interest to disclose.

## AUTHOR CONTRIBUTIONS

M.I. wrote the manuscript and designed the study. R.K., K.H., H.S., R.H., I.O., K.M., K.T., S.S., and T.T. proofread the manuscript.

## CONSENT

Written informed consent was obtained from the patient for publication of this Case Report and any accompanying images. A copy of the written consent is available for review by the editor in chief of this journal.

## DATA AVAILABILITY STATEMENT

The data that support the findings of this study are available from the corresponding author upon reasonable request.
